# Inspiratory muscle activation increases with COPD severity as confirmed by non-invasive mechanomyographic analysis

**DOI:** 10.1371/journal.pone.0177730

**Published:** 2017-05-18

**Authors:** Leonardo Sarlabous, Abel Torres, José A. Fiz, Juana M. Martínez-Llorens, Joaquim Gea, Raimon Jané

**Affiliations:** 1 Institute for Bioengineering of Catalonia, Barcelona, Spain; 2 Center for Biomedical Research Network of Bioengineering, Biomaterials and Nanomedicine, Barcelona, Spain; 3 Department of Automatic Control, Universitat Politècnica de Catalunya—BarcelonaTech (UPC), Barcelona, Spain; 4 Department of Respiratory Medicine, Hospital del Mar–IMIM. DCEXS, UPF. CIBERES, ISCiii. Barcelona, Spain; Telethon Institute for Child Health Research, AUSTRALIA

## Abstract

There is a lack of instruments for assessing respiratory muscle activation during the breathing cycle in clinical conditions. The aim of the present study was to evaluate the usefulness of the respiratory muscle mechanomyogram (MMG) for non-invasively assessing the mechanical activation of the inspiratory muscles of the lower chest wall in both patients with chronic obstructive pulmonary disease (COPD) and healthy subjects, and to investigate the relationship between inspiratory muscle activation and pulmonary function parameters. Both inspiratory mouth pressure and respiratory muscle MMG were simultaneously recorded under two different respiratory conditions, quiet breathing and incremental ventilatory effort, in 13 COPD patients and 7 healthy subjects. The mechanical activation of the inspiratory muscles was characterised by the non-linear multistate Lempel–Ziv index (MLZ) calculated over the inspiratory time of the MMG signal. Subsequently, the efficiency of the inspiratory muscle mechanical activation was expressed as the ratio between the peak inspiratory mouth pressure to the amplitude of the mechanical activation. This activation estimated using the MLZ index correlated strongly with peak inspiratory mouth pressure throughout the respiratory protocol in both COPD patients (r = 0.80, p<0.001) and healthy (r = 0.82, p<0.001). Moreover, the greater the COPD severity in patients, the greater the level of muscle activation (r = -0.68, p = 0.001, between muscle activation at incremental ventilator effort and FEV_1_). Furthermore, the efficiency of the mechanical activation of inspiratory muscle was lower in COPD patients than healthy subjects (7.61±2.06 vs 20.42±10.81, respectively, p = 0.0002), and decreased with increasing COPD severity (r = 0.78, p<0.001, between efficiency of the mechanical activation at incremental ventilatory effort and FEV_1_). These results suggest that the respiratory muscle mechanomyogram is a good reflection of inspiratory effort and can be used to estimate the efficiency of the mechanical activation of the inspiratory muscles. Both, inspiratory muscle activation and inspiratory muscle mechanical activation efficiency are strongly correlated with the pulmonary function. Therefore, the use of the respiratory muscle mechanomyogram can improve the assessment of inspiratory muscle activation in clinical conditions, contributing to a better understanding of breathing in COPD patients.

## Introduction

The force generated by the respiratory muscles is usually estimated from measurements of pressure. However, the relationship between force and pressure is complex [1]. This complexity may be closely related to the thoracic geometry which plays a major role in how efficiently force is converted into pressure. In patients with chronic obstructive pulmonary disease (COPD) the pulmonary hyperinflation leads to diaphragm shortening and deleterious changes in the muscle force-length relationship resulting in reduced force-generating capacity [2,3]. Thus, inspiratory muscle strength, endurance and/or mechanical efficiency may be reduced in such patients [4–6]. Inspiratory mouth pressure is easy to measure, both for the operator and subjects, and provides a global index of synergistic respiratory muscle action. However, the measurement of mouth pressure does not discriminate between weakness in the different respiratory muscles [1]. There is a lack of instruments for assessing respiratory muscle effort during the breathing cycle in clinical conditions. This shortfall has motivated the search for alternative non-invasive techniques for evaluating the mechanical activation of respiratory muscles and the efficiency of these.

Diaphragm electromyographic (EMG) signals recorded using an oesophageal electrode and transdiaphragmatic pressures obtained from oesophageal and gastric probes offer the most direct measurements of the respiratory drive in humans [7,8]. However, the complexity of these recordings and their invasive nature has prevented them being used routinely in clinical settings [9]. The level and pattern of respiratory muscle activation can be assessed non-invasively from surface EMG and mechanomyographic (MMG) signals, which are related to electrical and mechanical muscle activity, respectively. Surface EMG recorded in the second intercostal space at the position of the parasternal or other intercostal muscles has been used as a non-invasive alternative for assessing neural respiratory drive [10–12]. Surface EMGs from parasternal intercostal muscles have been shown to reflect lung disease severity in COPD and cystic fibrosis [13], and provide a sensitive measure of respiratory system load-capacity balance [14].

MMG signals provide complementary information and are the mechanical counterpart to EMG [15,16]. The MMG signal is the recording of the muscle surface oscillations caused by the mechanical activity of the motor units. These vibrations have been used to describe changes in motor unit recruitment, determine the onset of mechanical muscle response, discriminate between muscle fibre types, and assess muscle fatigue [17–19]. Since it is a mechanical signal, the MMG is not influenced by changes in skin impedance due to sweating, the placement of the MMG sensor does not have to be as precise as for a surface EMG, and the MMG does not suffer any bioelectrical interference from adjacent muscles, heart activity, or nerve stimulation [16]. Previous studies have compared the transdiaphragmatic pressure and diaphragm muscle compound motor action potential (CMAP) against the diaphragmatic MMG signal (also known as the acoustic signal or phonomyography of the diaphragm) [20–23], with the aim of evaluating the mechanical response of the diaphragm evoked by phrenic nerve stimulation. These studies have been key when considering the MMG as a promising research tool for evaluating respiratory muscle strength, as it is technically easy and non-invasive [1].

Recently, respiratory muscle MMG signals acquired using accelerometers positioned over the lower chest wall were used to non-invasively record the mechanical activation of inspiratory muscles (diaphragm and intercostal muscles) during breathing in animal models (dogs) [24,25], healthy subjects [26] and COPD patients [27,28]. This recording position is proximal to the apposition zone of the diaphragm muscle. This muscle is therefore likely to be the main contributor to the MMG signal, although the intercostal muscles of the lower chest wall may also contribute. The respiratory muscle MMG signal could indicate the degree of mechanical activation of inspiratory muscles [26], and could be used as a sensitive measure of respiratory system load-capacity balance comparable to surface EMGs from the parasternal intercostal muscles [14].

Typically, the root mean square (RMS) has been used as the classical parameter for estimating MMG amplitude [15,29,30]. However, RMS is greatly affected by various types of noise such as motion artefacts caused by breathing, impulsive noise, spurious spikes, and cardiac vibration interference. To overcome these types of noise, our group proposed the use of the non-linear multistate Lempel–Ziv index (MLZ) [24]. The MLZ index is an improved version of the Lempel–Ziv algorithm [31] for quantifying the impact of complexity changes on amplitude variations of discrete time signals, using fixed thresholds and more than two quantisation levels. Thus, MLZ gives more weight to the contribution of the high-complexity components of a signal, while being less affected by the non-random components. In [24] we found that the MLZ of respiratory muscle MMG recorded in dogs positively correlated with changes in the amplitude components, but was less affected by non-random components like structured impulsive noise. We also found that the MLZ correlated more strongly with mouth pressure than classical amplitude parameters, such as average rectified value or RMS.

The main objective of this study was to estimate the mechanical activation of the inspiratory muscles of the lower chest wall (diaphragm and intercostal muscles) using the MLZ of MMG obtained during quiet breathing as well as during an incremental ventilatory effort. A secondary objective was to evaluate the efficiency of this mechanical activation, expressed as the ratio between the peak inspiratory mouth pressure at tidal volume and the MLZ of MMG. Changes in inspiratory mouth pressure may give a reasonable approximation of the overall mechanical output of the synergistic respiratory muscles and the distensibility of the system [1], whereas changes in the MLZ of MMG could be related to the inspiratory muscle effort necessary to produce this mechanical output. Finally, the potential relationship between mechanical activation and efficiency with pulmonary function in COPD and healthy subjects was evaluated. This novel approach is aimed at improving the non-invasive assessment of respiratory muscle activation in COPD patients.

## Materials and methods

### Subjects

The current study involved 20 subjects, 13 patients (2 female and 11 male) with moderate-to-very severe COPD according to the ATS/ERS criteria [32] and 7 healthy subjects (3 female and 4 male) (Table 1). The study was approved by the Local Ethics Committee (Hospital del Mar-IMIM, Barcelona, Spain), conducted in accordance with the declaration of Helsinki for studies in humans, and written informed consent was obtained in all cases.

**Table 1:** General and functional data.

	COPD patients (n = 13)	Healthy subjects (n = 7)	p-value
Sex female/male	2/11	3/4	-
Age, yrs	68 ± 9	50 ± 20	0.04
BMI, kg/m^2^	23.5 ± 4	26.6 ± 4	NS
**Lung function**			
FEV_1,_ % pred.	37 ± 15	106 ± 14	0.0002
FVC, % pred.	59 ± 14	99 ± 10	0.0002
FEV_1_/FVC, %	45 ± 10	78 ± 4	0.0002
RV/TLC, %	70 ± 8	35 ± 5	0.0001
DLco, % pred.	53 ± 21	93 ± 7	0.0005
**Respiratory protocol**			
Maximum IP_peak,_ cmH_2_O	3.6 ± 1	8 ± 5	0.002
Duration, s	305 ± 59	194 ± 139	0.009
Duty Cycles	115 ± 23	75 ± 50	NS
RR at QB	18.8 ± 4	19.9 ± 8	NS
RR at IVE	53 ± 20	36.7 ± 22	0.02

Data are presented as mean±SD. Abbreviations: BMI: body mass index; FEV_1_: forced expiratory volume in one second; FVC: forced vital capacity; RV: residual volume; TLC: total lung capacity; DLco: carbon monoxide diffusing capacity; Maximum IP_peak_: maximum value of the peak inspiratory mouth pressure reached during the protocol; Duration: duration of the incremental flow protocol; Duty Cycles: number of cycles performed during the incremental breathing protocol. RR: respiratory rate (breaths/minute); QB: quiet breathing; IVE: incremental ventilatory effort. % pred: % predicted.

### Measurements

Inspiratory mouth pressure was recorded using a pressure transducer (Digima Premo 355, Special Instruments, Noerdlingen, Germany), connected to a recording system (MP100, Biopac Systems, Inc, Goleta, CA, USA). The respiratory muscle MMG signal was simultaneously recorded using two capacitive accelerometers (8312B2, Kistler, Winterthur, Switzerland), with a frequency response of 0–250 Hz. The accelerometers were attached to the skin with double-sided adhesive tape, on the chest surface, over the seventh and eighth intercostal spaces at the left and right anterior axillary lines, respectively. This position is proximal to the apposition zone of the diaphragm muscle in order to better register its mechanical vibrations.

The inspiratory mouth pressure and respiratory muscle MMG signals were recorded at a sampling rate of 2000 Hz using a 12-bit analogue-to-digital converter, and decimated at a sampling rate of 200 Hz. The MMG recordings were filtered through a zero-phase fourth-order Butterworth filter with a band pass from 5 to 25 Hz to eliminate the low frequency movement of the chest wall caused by respiration.

### Respiratory protocol

Respiratory signals were recorded with the subject sitting upright in a comfortable position, wearing a nose clip, and breathing through a mouthpiece connected to a T-tube. Firstly, the subjects were asked to breathe normally for one minute in order to evaluate their inspiratory mechanical activation during quiet breathing (QB). Subsequently, they were coached in how to perform an incremental ventilatory effort (IVE) protocol. To do this, an experienced physician instructed the subjects to regulate their respiration, progressively and controllably increasing the rhythm and intensity of their breathing from shallow to the deepest breaths they were able to take. Then, they progressively decreased the rhythm and intensity of their breathing until they once again reached shallow breathing [33]. In this way, a wide range of inspiratory pressures without occlusion could be analysed, depending on the total airflow range of each subject. This procedure was repeated three times per subject to ensure both reproducibility and maximum effort. The total duration of the protocol ranged from 2 to 6 minutes.

### Multistate Lempel–Ziv analysis

The mechanical activity of the inspiratory muscles was estimated using the non-linear multistate Lempel–Ziv (MLZ) index, which was compared with an estimation obtained using classical RMS. The MLZ index is a new version of the Lempel–Ziv algorithm [31], which calculates the complexity of a discrete-time signal using fixed thresholds and more than two quantisation levels. MLZ measures the impact of complexity changes on the amplitude variations of random signal components. Two principal parameters are required to calculate the MLZ: the quantisation step size, referring to the size of the fixed thresholds, and the number of quantisation levels. The MLZ index is more robust against impulsive noise caused by diaphragm movement than RMS. A more detailed explanation of MLZ behaviour can be found in [24].

### Respiratory signal analysis

The overall mechanical output of the inspiratory muscles during breathing was determined for each respiratory cycle by measuring the absolute value of the peak inspiratory mouth pressure (IP_peak_) during tidal volume (without occlusion). IP_peak_ is a combination of both the mechanically expressed inspiratory muscle activity and the distensibility of the system. The mechanical inspiratory muscle performance was determined by measuring the MLZ index and RMS of the average of the left and right respiratory MMG signals (MMG-MLZ and MMG-RMS, respectively). The averaged respiratory MMG signal provides the overall contribution to the mechanical activation made by the inspiratory muscles from the two hemidiaphragms. To calculate the MLZ index a quantisation step size of 0.24 was used with 50 quantisation levels [24].

The IP_peak_, MMG-MLZ and MMG-RMS indices were obtained and analysed for each inspiratory cycle using the inspiratory time during both QB and IVE. The IVE cycles were selected as the 20 per cent of the cycles of the incremental ventilatory effort with the highest IP_peak_. Assessments of inspiratory muscle effort and mechanical activation were obtained using the median value of IP_peak_ and both MMG indices, respectively.

### Efficiency of the mechanical activation of inspiratory muscles

The ratio between IP_peak_ and the MMG amplitude estimated by MLZ was used as an expression of inspiratory muscle mechanical activation efficiency (E_MMG_-MLZ) [27]. This ratio indicates the level of activity/effort of inspiratory muscles necessary to achieve a specific mechanical outcome. The E_MMG_-MLZ was then compared to the alternative estimation of inspiratory muscle efficiency obtained from the IP_peak_ to MMG amplitude estimated by the RMS (E_MMG_-RMS) ratio. This comparison (E_MMG_-MLZ *versus* E_MMG_-RMS) was analysed under QB and IVE conditions.

### Statistical analysis

The relationship between the IP_peak_ and MMG amplitude was assessed using the Pearson correlation coefficient (r). For this analysis all the respiratory cycles in the protocol were considered. The potential relationships between lung function and the variables derived from inspiratory muscles (IP_peak_, MMG amplitude and estimations of mechanical efficiency) were also analysed under QB and IVE conditions using the r coefficient. The differences in the mean of each derived inspiratory muscle variable between the COPD and healthy groups under the same respiratory conditions were assessed using the Mann-Whitney U test. Furthermore, the mean values of each inspiratory muscle variable under QB and IVE conditions were compared using the Wilcoxon signed-rank test in the overall population; p-values of <0.05 were considered significant.

## Results

**Fig 1:**
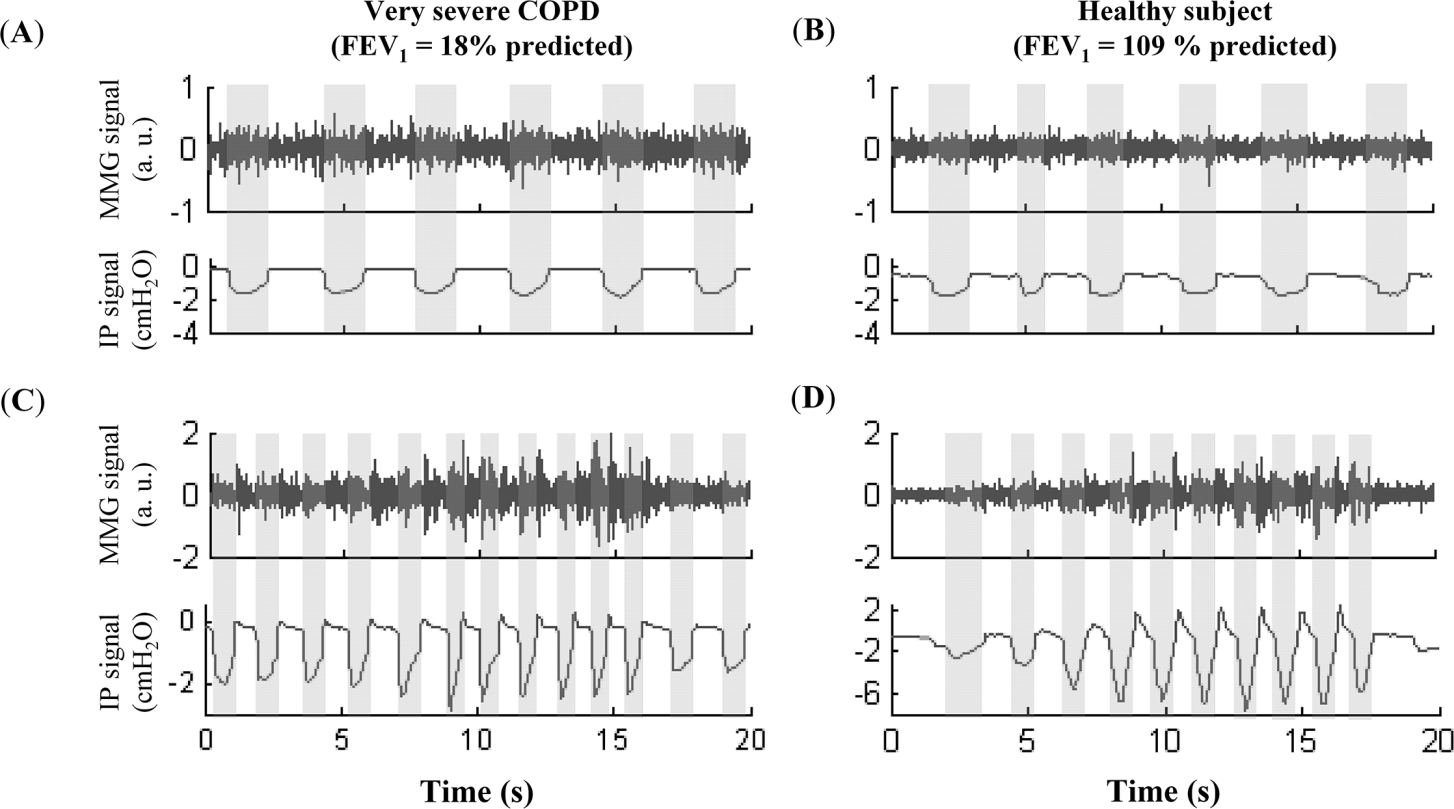
Signals recorded during the respiratory protocol. Representative traces of the mechanomyogram (MMG) signal and mouth inspiratory pressures (IP) at quiet breathing (graphs **A** and **B**) and during incremental ventilatory effort (graphs **C** and **D**) in a very severe COPD patient (graphs **A** and **C**) and in a healthy subject (graphs **B** and **D**).

The general and lung function data of the subjects is summarised in Table 1. Representative traces of both mouth pressure and MMG at QB and IVE in a very severe COPD patient and a healthy subject are shown in Fig 1. The subjects showed a mean respiratory frequency of 19.2±5 breaths per minute at QB (range 12 to 36), with no significant differences between COPD and healthy subjects (18.8±4 *versus* 19.9±8, respectively); and 47.3±22 cycles per minute during IVE (range 25 to 95), with significantly higher values in COPD than healthy subjects (53.0±20.0 *versus* 36.6±22.0, respectively). No significant correlations were observed between the subjects’ general data and the MMG amplitude (scatter plots and Pearson correlation coefficients of these variables are shown in S1 File).

### IP_peak_ versus inspiratory muscle mechanical activation indices

**Fig 2:**
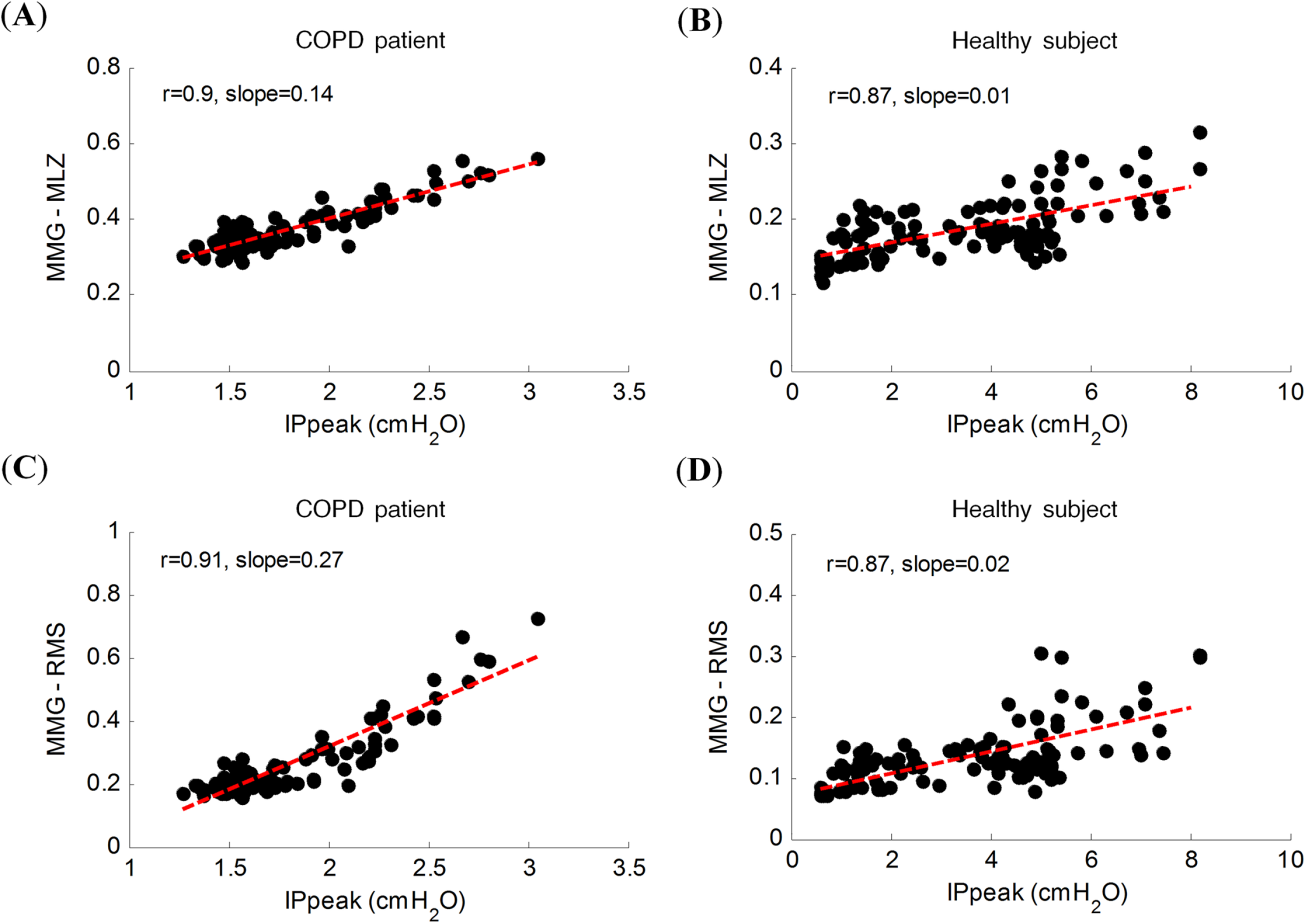
Absolute value of respiratory parameters throughout the progressive respiratory protocol. Scatter plot of the absolute peak inspiratory mouth pressure values (IP_peak_), the amplitude of the mechanical activation of the inspiratory muscles estimated by multistate Lempel–Ziv (MMG-MLZ) (graphs **A** and **B**) and the root mean square (MMG-RMS) (graphs **C** and **D**) in a very severe COPD patient (graphs **A** and **C**) and a healthy subject (graphs **B** and **D**), throughout the progressive respiratory protocol. All *r* values are significant at p<0.001.

IP_peak_ and the two MMG indices increased with the ventilatory effort. The correlation analysis showed significant positive r values (Table 2). There were no significant differences in the correlation values between the COPD and healthy groups. Scatter plots of the IP_peak_ and MMG indices for two representative subjects, one COPD patient and one healthy subject, are shown in Fig 2.

**Table 2:** Correlation between IP_peak_ and MMG indices.

Groups	IP_peak_ *versus* MMG-MLZ	IP_peak_ *versus* MMG-RMS
COPD patients	0.80 ± 0.21	0.78 ± 0.24
Healthy subjects	0.82 ± 0.17	0.85 ± 0.11
Total		
Mean ± SD	0.81 ± 0.19	0.80 ± 0.2

IP_peak_: Peak inspiratory mouth pressure at tidal volume; MLZ: Multistate Lempel-Ziv; RMS: root mean square. All *r* values are significant at p<0.001.

### Mechanical activation performance of inspiratory muscles during QB and IVE

**Fig 3:**
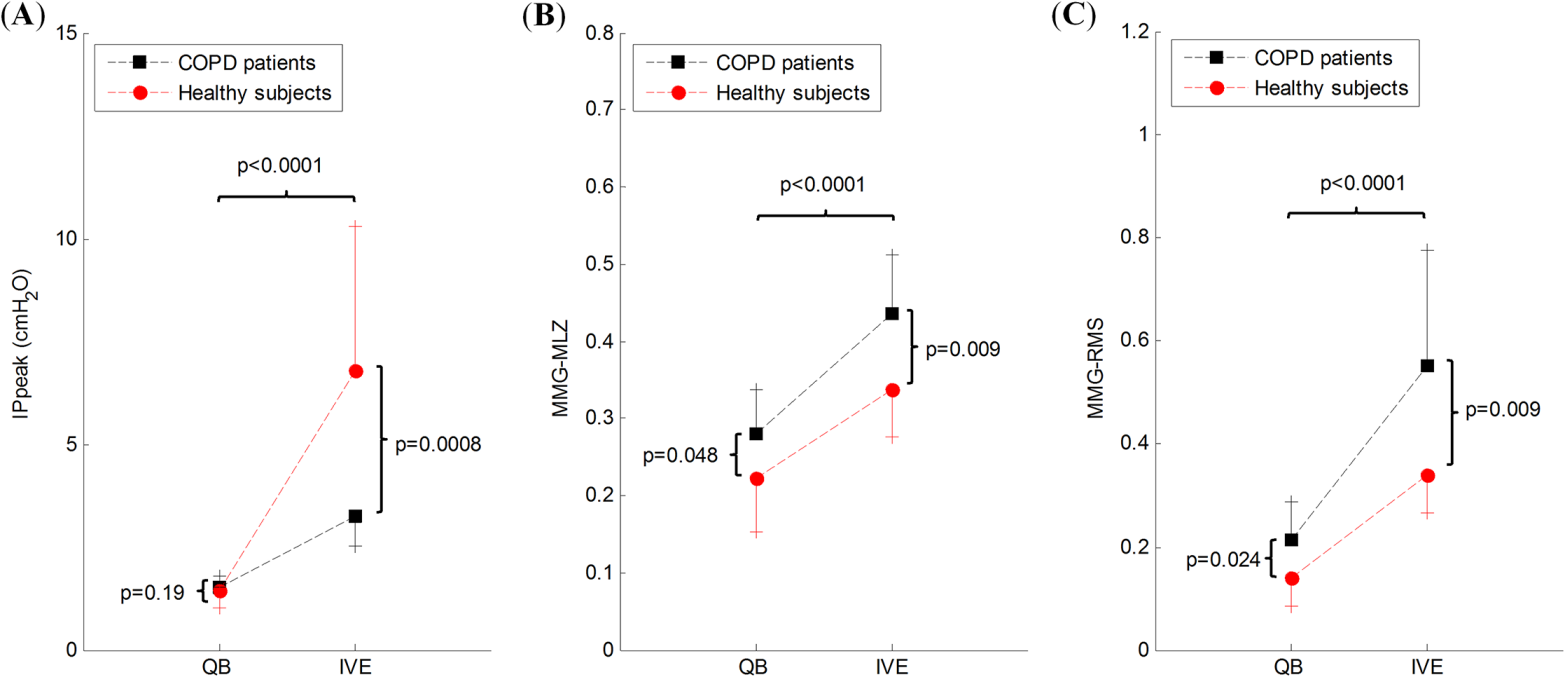
Value of mechanical activation measures at different ventilation levels. Representation (mean and SD) of (**A**) IP_peak_, (**B**) MMG-MLZ, and (**C**) MMG-RMS, in COPD patients and healthy subjects. QB: quiet breathing; IVE: incremental ventilatory effort.

Fig 3 shows the values of IP_peak_, MMG-MLZ and MMG-RMS at QB and during IVE in both the COPD and healthy groups. IP_peak_, MMG-MLZ and MMG-RMS increase significantly with the level of ventilation in both groups. However, no differences were observed between the two groups for IP_peak_ at QB, while significant differences were seen at IVE. Nevertheless, MMG-MLZ was significantly higher in the COPD group than in the healthy group at both QB and IVE indicating greater muscle activation. MMG-RMS showed comparable behaviour to MMG-MLZ, with a similar significance value at IVE and a higher significance value at QB.

### Relationship between muscle activation and pulmonary function

The analysis revealed significant correlations between lung function and IP_peak_ at IVE (Table 3). Both indices of the mechanical activation of inspiratory muscles, MMG-MLZ and MMG-RMS, correlated significantly with lung function at both QB and IVE. Estimating the mechanical activation of inspiratory muscles using RMS showed a higher correlation with lung function at QB, while MLZ showed a higher correlation with lung function at IVE. Interestingly, the correlation values between lung function and the mechanical activation of inspiratory muscles estimated by MLZ at IVE were higher than those reported by IP_peak_ for FVC, FEV_1_/FVC and DLco, while being comparable for FEV_1_ and RV/TLC.

**Table 3:** Correlations between pulmonary function and IP_peak_ and both inspiratory muscle mechanical activation indices.

Lung function	IP_peak_		MMG-MLZ		MMG-RMS	
	r value	p value	r value	p value	r value	p value
**QB**						
FEV_1_, % pred.	- 0.19	NS	- 0.61	0.004	- 0.66	0.001
FVC, % pred.	- 0.18	NS	- 0.66	0.002	- 0.72	< 0.001
FEV_1_/FVC, %	- 0.14	NS	- 0.61	0.004	- 0.66	0.002
RV/TLC, %	0.02	NS	0.56	0.01	0.62	0.005
DLco, % pred.	- 0.11	NS	- 0.65	0.002	- 0.71	< 0.001
**IVE**						
FEV_1_, % pred.	0.71	< 0.001	- 0.68	< 0.001	- 0.60	< 0.01
FVC, % pred.	0.69	< 0.001	- 0.74	< 0.001	- 0.65	0.002
FEV_1_/FVC, %	0.66	0.002	- 0.68	0.001	- 0.59	< 0.01
RV/TLC, %	- 0.69	0.001	0.68	0.002	0.59	< 0.01
DLco, % pred.	0.56	< 0.05	- 0.86	< 0.001	- 0.76	< 0.001

IP_peak_: peak inspiratory mouth pressure; MLZ: Multistate Lempel-Ziv; RMS: root mean square; QB: quiet breathing; IVE: incremental ventilatory effort; FEV_1_: forced expiratory volume in one second; FVC: forced vital capacity; FEV_1_/FVC: proportion of the forced vital capacity exhaled in the first second; RV: residual volume; TLC: total lung capacity; DLco: carbon monoxide diffusing capacity; % pred: % predicted. NS: not significant.

### Efficiency of the mechanical activation of inspiratory muscles

The E_MMG_-MLZ of the healthy group was 6.72±1.73 and 20.42±10.81, for QB and IVE, respectively (Table 4). These values were significantly higher (p-value = 0.048 and p-value = 0.0002, respectively) than those observed in COPD patients (5.57±1.51 and 7.61±2.06, for QB and IVE, respectively). Interestingly, although the E_MMG_-MLZ values of both groups rose in a similar way as the respiratory effort increased, this was not the case for E_MMG_-RMS, which decreased significantly during the breathing protocol in the COPD group, 7.76±2.82 *versus* 6.69±2.95, but increased in the healthy group 11.1±3.03 *versus* 20.57±11.47, for QB and IVE, respectively. Despite significant differences between the two groups for E_MMG_-RMS at both QB and IVE, there were no significant differences when the level of ventilation increased from QB to IVE, if all subjects are taken into account.

**Table 4:** Efficiency of the mechanical activation of inspiratory muscles.

Groups	E_MMG_-MLZ		E_MMG_-RMS	
	QB	IVE	QB	IVE
COPD patients	5.57 ± 1.51	7.61 ± 2.06	7.76 ± 2.82	6.69 ± 2.95
Healthy subjects	6.72 ± 1.73	20.42 ± 10.81	11.1 ± 3.03	20.57 ± 11.47
p-value, COPD vs Healthy	0.048	0.0002	0.013	0.0003
p-value, QB vs IVE (all subjects)	< 0.0001		NS	

E_MMG_-MLZ: Efficiency of mechanical activation of inspiratory muscles estimated by Multistate Lempel-Ziv; E_MMG_-RMS: efficiency of mechanical activation of inspiratory muscles estimated by root mean square; QB: quiet breathing; IVE: incremental ventilatory effort.

### Correlation between pulmonary function and muscle efficiency

The correlation between inspiratory muscle mechanical activation efficiency and lung function parameters was moderate-to-very strong (Table 5). The highest correlation values for E_MMG_-MLZ and E_MMG_-RMS at QB were observed with FVC and DLco, while at IVE the highest correlation values were observed with RV/TLC and DLco.

**Table 5:** Correlations between pulmonary function and inspiratory muscle efficiency.

Lung function	E_MMG_-MLZ		E_MMG_-RMS	
	r value	p value	r value	p value
**QB**				
FEV_1_, % pred	0.43	0.06	0.63	0.003
FVC, % pred	0.49	< 0.05	0.70	< 0.001
FEV_1_/FVC, %	0.45	< 0.05	0.64	0.002
RV/TLC, %	- 0.46	< 0.05	- 0.58	< 0.01
DLco, % pred	0.49	< 0.05	0.71	< 0.001
**IVE**				
FEV_1_, % pred	0.78	< 0.001	0.78	< 0.001
FVC, % pred	0.77	< 0.001	0.78	< 0.001
FEV_1_/FVC, %	0.73	< 0.001	0.74	< 0.001
RV/TLC, %	- 0.85	< 0.001	- 0.84	< 0.001
DLco, % pred	0.81	< 0.001	0.86	< 0.001

MLZ: Multistate Lempel-Ziv; RMS: root mean square; QB: quiet breathing; IVE: incremental ventilatory effort; FEV_1_: forced expiratory volume in one second; FVC: forced vital capacity; FEV_1_/FVC: proportion of the forced vital capacity exhaled in the first second; RV: residual volume; TLC: total lung capacity; DLco: carbon monoxide diffusing capacity; % pred: % predicted. NS: not significant.

Lung function parameters correlated better with the efficiency indices (E_MMG_-MLZ and E_MMG_-RMS) than with inspiratory pressures or activation indices (MMG-MLZ and MMG-RMS) during IVE. The correlations of E_MMG_-MLZ and E_MMG_-RMS increased in parallel to the respiratory effort.

## Discussion

The main contribution of the present study is the proposal of a new non-invasive method for estimating the mechanical activity and efficiency of inspiratory muscles during breathing. This method is based on a non-linear index (MMG-MLZ) calculated from the respiratory MMG, using IP_peak_ as the reference parameter. The peak and mean inspiratory mouth pressures at tidal volume provide a reasonable approximation of the muscular effort during the inspiration. In addition, the IP_peak_ also includes respiratory system compliance and resistance components. Both inspiratory mouth pressure parameters could be used as a global reference index for synergistic respiratory muscle action. In this study, no significant differences were observed in the relationship between both peak and mean inspiratory mouth pressures and the mechanical activity and efficiency of inspiratory muscles during breathing, or pulmonary function in COPD (S2 File).

The respiratory MMG signal is predominantly produced by the diaphragm muscle but also integrates contributions from lower chest wall muscles, making it an alternative method for evaluating respiratory muscle effort generated during breathing [24,26,27]. The strong linear relationship between IP_peak_ and the respiratory MMG is in agreement with the results obtained previously from other striated muscles [34,35], where researchers found a positive correlation between the force produced by these and their MMG amplitude. Our results show that this linear relationship tends to be stronger in the healthy group, who achieved a greater IP_peak_ increment throughout the protocol than CODP patients. The non-linear MMG-MLZ-derived parameter strongly correlated with IP_peak_ in both groups, reinforcing the robustness of this index for estimating increments of mechanical activation in inspiratory muscles. This closely agrees with our previous results obtained using an animal model [24].

Moreover, the mechanical activation of the inspiratory muscles calculated using the MLZ index correlated strongly with lung function during QB but not as much as RMS, while during IVE the MLZ index correlated more strongly than RMS and was comparable to IP_peak_. The higher levels of MMG in COPD patients are likely to be the result of two important factors. They probably indicate that these indices better reflect the ventilatory effort rather than the respiratory pressure (the final outcome of this effort). In fact, during QB and IVE they were closely related, respectively, to both pulmonary hyperinflation and DLco (two very well-known functional markers of emphysema). The first of these induces a flattening and shortening of the diaphragm, which becomes far from its optimal contracted length. The significant correlations also observed with spirometric parameters probably reflect the effort involved in fighting resistive and threshold loads derived from airway obstruction, which lead to a recruitment of additional motor units and/or an increase in the motor unit firing rate. It should be noted that these correlations were, in general, slightly stronger with MMG-MLZ than with MMG-RMS.

The baseline values of IP_peak_ and both indices of mechanical activation of the inspiratory muscles were higher in COPD patients than in healthy subjects (Fig 3) and increased in response to the respiratory effort (i.e., QB *versus* IVE). The increase in IP_peak_ at QB was not significant different between COPD and healthy subjects, while at IVE this was significantly higher in the healthy group. This probably indicates the fact that although the muscles are more mechanically activated in COPD patients during ventilatory efforts, their inspiratory muscle mechanical activation efficiency is lower, most likely due to pulmonary hyperinflation [5]. This inference is also suggested by the strong correlations obtained between inspiratory muscle efficiency indices (E_MMG_-MLZ and E_MMG_-RMS) and lung function parameters.

The present results suggest that individuals with COPD need greater mechanical activation of inspiratory muscles to achieve similar pressures during ventilatory efforts than healthy subjects. In addition, both E_MMG_-MLZ and E_MMG_-RMS correlated strongly with pulmonary function during IVE (Table 5). The MLZ index quantifies the impact of complexity changes on the amplitude variations of random signal components [24]. The robustness of MLZ against non-random components makes it a more reliable option for assessing the muscle activity derived from respiratory MMG signals than other classical linear indices such as RMS. This study not only confirms the robustness of the MLZ index as a good estimator of the mechanical activation of inspiratory muscles strongly correlated with pulmonary function, but also reinforces its potential for estimating the efficiency of the mechanical activation.

Interestingly, the moderate-to-very strong correlation observed between the efficiency indices and lung function (Table 5) was much higher than that observed between the latter and IP_peak_, MMG-MLZ or MMG-RMS (Table 3) for IVE. Our results suggest that the E_MMG_-MLZ index could even be used as a marker for inspiratory muscle activity in COPD in a clinical setting.

The present study does have, however, certain limitations. Firstly, it is a pilot study with a relatively small sample size that could limit the generalisation of the results. Nevertheless, this sample size was large enough to yield statistically significant results using non-parametric statistical methods. Furthermore, the present findings are consistent with previous studies by our own group using an animal model [24,25], healthy subjects [26], and COPD patients [27,28]. A second limitation is the fact that, despite the patients being guided by appropriately-trained and knowledgeable staff, the present study does not quantify whether or not the subjects performed maximal and similar efforts to achieve the theoretical maximum voluntary ventilation. Nevertheless, the progressive respiratory manoeuvre used in this study allows an important level of ventilatory effort to be achieved in a way that is comfortable for patients with severe and very severe cases of the disease. A third limitation is that the greater MMG activity observed in COPD patients could be affected by the significant shortening of the diaphragm muscle and the length of its zone of apposition against the chest wall [36]. This different diaphragm configuration implies changes in the length and characteristics of the tissue lying between the diaphragm muscle and the MMG sensor that could affect the characteristics of the MMG signal [37,38]. Finally, in this study the mechanical activity of the respiratory muscles estimated with the respiratory MMG signal has been compared with the peak inspiratory mouth pressure during tidal volume in an unoccluded airway. These pressure values combine both the mechanical response of the inspiratory muscles and the distensibility of the system, but are low due to the dissipation of pressure with flow and do not directly reflect the alveolar pressure.

## Conclusions

The present results indicate that the mechanical activation of the inspiratory muscles is better reflected by the non-linear MLZ index, which correlates more strongly with lung function than IP_peak_ at QB but not as well as RMS, while at IVE the MLZ index correlated more strongly than RMS and was comparable to IP_peak_. Moreover, the efficiency of the inspiratory muscle activation, expressed as the ratio between the mechanical outcome and the level of inspiratory muscle effort, is better reflected by the MLZ than the RMS index, and clearly decreases with the severity of the disease. This study highlights the potential of respiratory MMG analysis for non-invasive studies of inspiratory muscle activity. In addition, this method could provide a better understanding of the mechanics of breathing in COPD patients.

## Supporting information

S1 File Relationship between anthropometric data and inspiratory muscle mechanical activation estimated using the MLZ and RMS indices. (PDF)

S2 File Relationship of the inspiratory mechanical activation and inspiratory muscle mechanical activation efficiency and pulmonary function using the mean inspiratory mouth pressure as a global reference index for synergistic respiratory muscle activity. (PDF)
