# Supplementary material for: Inspiratory muscle activation increases with COPD severity as confirmed by non-invasive mechanomyographic analysis
Source: PLoS One. 2017 May 18;12(5):e0177730. doi: 10.1371/journal.pone.0177730 (PMC5436747; doi:10.1371/journal.pone.0177730)
Supplement: S1 File — (PDF) [file pone.0177730.s001.pdf]

S1 File. Relationship between anthropometric data and inspiratory muscle mechanical activation estimated using the MLZ and RMS indices.

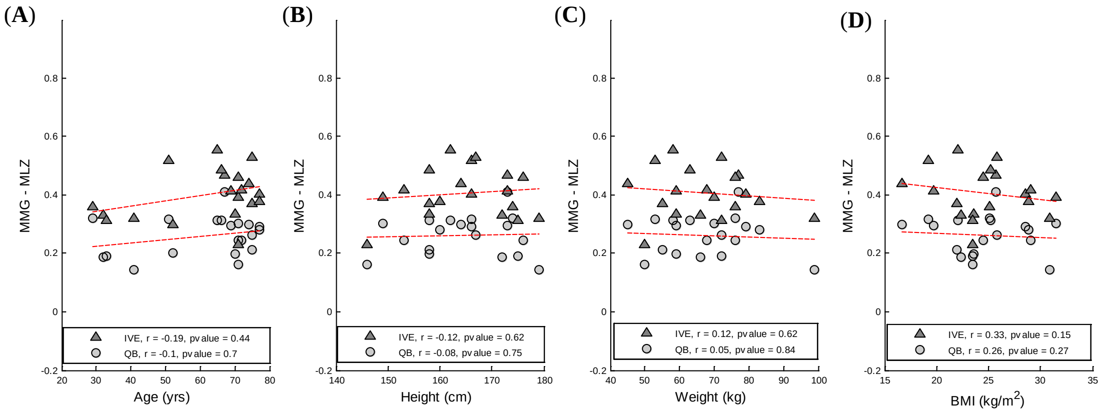

S1 Fig. Pearson correlation coefficient between anthropometric data and the Multistate Lempel-Ziv (MLZ) index of respiratory MMG signal. Relationship during quiet breathing (QB) and incremental ventilatory effort (IVE).

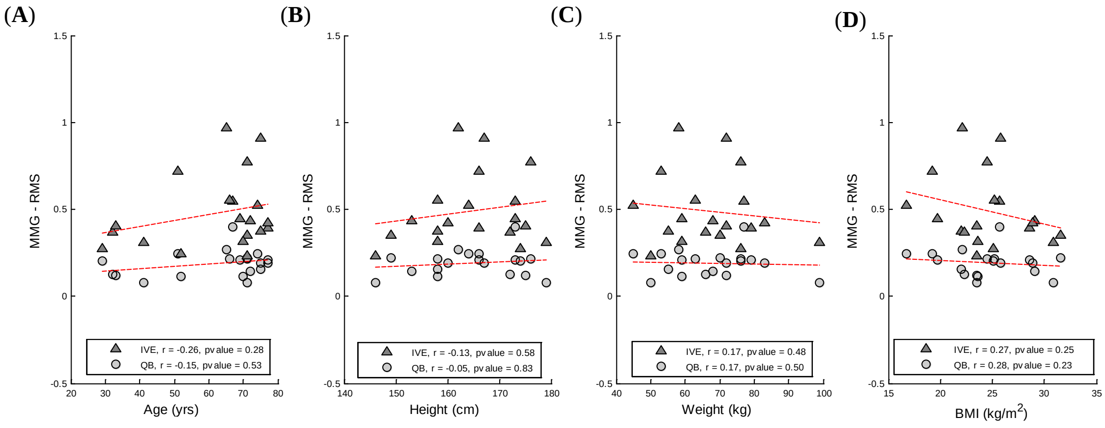

S2 Fig. Pearson correlation coefficient between anthropometric data and the RMS of respiratory MMG signal. Relationship during QB and IVE.

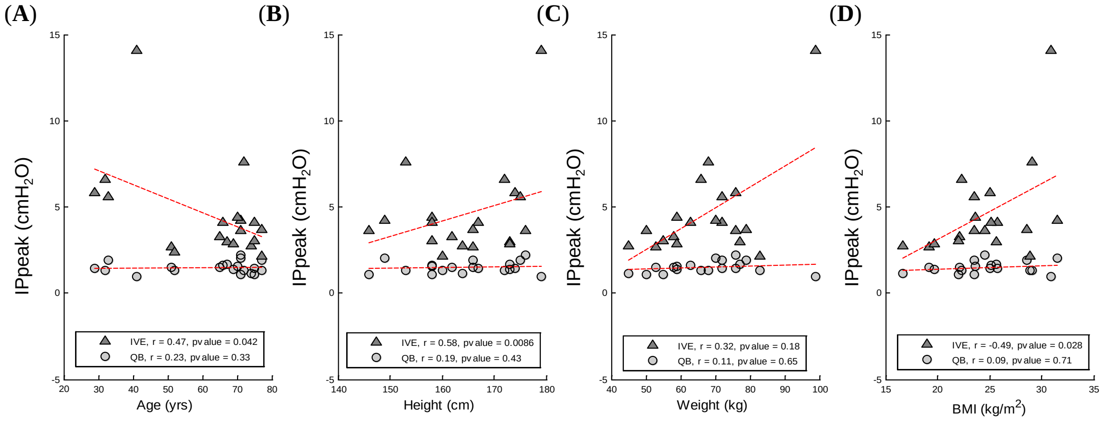

S3 Fig. Pearson correlation coefficient between anthropometric data and the peak inspiratory mouth pressure (IPpeak). Relationship during QB and IVE.
