# Supplementary material for: Inspiratory muscle activation increases with COPD severity as confirmed by non-invasive mechanomyographic analysis
Source: PLoS One. 2017 May 18;12(5):e0177730. doi: 10.1371/journal.pone.0177730 (PMC5436747; doi:10.1371/journal.pone.0177730)
Supplement: S2 File — (PDF) [file pone.0177730.s002.pdf]

S2 File. Relationship of the inspiratory mechanical activation and inspiratory muscle mechanical activation efficiency and pulmonary function using the mean inspiratory mouth pressure as a global reference index for synergistic respiratory muscle activity. Mean inspiratory pressure (IPmean) instead of peak inspiratory pressure (IPpeak).

The peak and mean inspiratory mouth pressures provide a reasonable approximation of the muscular effort throughout the inspiration. In addition the maximum inspiratory mouth pressure also includes respiratory system compliance and resistance components. Both parameters could be used as a global reference indices for synergistic respiratory muscles action. In this study no significant differences were observed in the relationship between both global indices with the mechanical activity and the efficiency of inspiratory muscles during breathing, and pulmonary function in COPD (see Tables and Figures of the original paper).

TABLE 2	Correlation (*r*) between IPmean and MMG indices	
**Groups**	*r* values (IPmean *versus* MMG-MLZ)	*r* values (IPmean *versus* MMG-RMS)
COPD subjects	0.76 ± 0.24	0.73 ± 0.26
Healthy subjects	0.82 ± 0.18	0.85 ± 0.13
Total		
Mean ± SD	0.78 ± 0.22	0.77 ± 0.23
IPmean: Mean inspiratory mouth pressure; MLZ: Multistate Lempel-Ziv; RMS: root mean square; All *r* values are significant at p<0.001.		

Observation 1: Correlation between IPmean and MMG-MLZ and MMG-RMS decreased slightly in some subjects regarding the correlation found between IPpeak and MM-MLZ and MMG-RMS, respectively (see Table 2 in the original paper).

TABLE 3	Correlations (r) between pulmonary function and IP_{mean} and both indices of mechanical activation of inspiratory muscles.					
Lung function	IPmean		MMG-MLZ		MMG-RMS	
	r value	p value	r value	p value	r value	p value
**QB**						
FEV_1, % pred	-0.23	NS	-0.61	0.004	- 0.66	0.0014
FVC, % pred	-0.20	NS	-0.66	0.002	- 0.72	0.0003
FEV_1/FVC, %	-0.19	NS	-0.61	0.004	- 0.66	0.0015
RV/TLC, %	0.02	NS	0.56	0.01	0.62	0.0050
DLco, % pred	-0.08	NS	-0.65	0.002	- 0.71	0.0007
**IVE**						
FEV_1, % pred	0.75	0.0001	-0.67	0.0012	- 0.60	0.0055
FVC, % pred	0.73	0.0002	-0.72	0.003	- 0.65	0.0018
FEV_1/FVC, %	0.69	0.0007	-0.66	0.014	- 0.59	0.0066
RV/TLC, %	-0.75	0.0002	0.66	0.0018	0.59	0.0073
DLco, % pred	0.63	0.0038	-0.84	< 0.0001	- 0.76	0.0001
IPmean: mean inspiratory mouth pressure; MLZ: Multistate Lempel-Ziv; RMS: root mean square; QB: quiet breathing; IVE: incremental ventilatory effort; FEV_1: forced expiratory volume in one second; FVC: forced vital capacity; FEV_1/FVC: proportion of the forced vital capacity exhaled in the first second; RV: residual volume; TLC: total lung capacity; DLco: carbon monoxide diffusing capacity; % pred: % predicted. NS: not significant.						

Quiet breathing (QB) cycles were considered only the respiratory cycles before the first incremental flow manoeuvre. The incremental ventilatory effort (IVE) cycles were selected as the 20 per cent of the cycles of the incremental flow respiratory test with the highest mean IP value (when analysing the IPpeak them the highest maximum value of IP is used).

Observation 2: Correlations between IPmean and lung function parameters have increased compared to the IPpeak, especially at IVE, which shows that almost all correlations are significant. Correlations between MMG parameters and lung function parameters are the same of the original paper (see Table 3).

TABLE 4	Correlations (r) between pulmonary function and efficiency of inspiratory muscles.			
Lung function	E_{MMG}-MLZ		E_{MMG}-RMS	
	r value	p value	r value	p value
**QB**				
FEV_1, % pred	0.39	0.087	0.57	0.0083
FVC, % pred	0.47	0.036	0.65	0.0018
FEV_1/FVC, %	0.41	0.069	0.58	0.0069
RV/TLC, %	-0.48	0.037	-0.56	0.0118
DLco, % pred	0.53	0.019	0.70	0.0008
**IVE**				
FEV_1, % pred	0.81	< 0.0001	0.82	< 0.0001
FVC, % pred	0.80	< 0.0001	0.82	< 0.0001
FEV_1/FVC, %	0.76	0.0001	0.78	0.0001
RV/TLC, %	-0.86	< 0.0001	-0.83	< 0.0001
DLco, % pred	0.83	< 0.0001	0.87	< 0.0001

MLZ: Multistate Lempel-Ziv; RMS: root mean square; QB: quiet breathing; IVE: incremental ventilatory effort; FEV_1: forced expiratory volume in one second; FVC: forced vital capacity; FEV_1/FVC: proportion of the forced vital capacity exhaled in the first second; RV: residual volume; TLC: total lung capacity; DLco: carbon monoxide diffusing capacity; % pred: % predicted.

Observation 3: In general, the correlation values for E_{MMG}-MLZ decreased slightly at QB, except for FVC and RV/TLC that increased, while at IVE the correlation increased for all cases. E_{MMG}-RMS showed comparable behaviour to E_{MMG}-MLZ (see Table 5 in the original paper).
